# The impact of microfluidics in high-throughput drug-screening applications

**DOI:** 10.1063/5.0087294

**Published:** 2022-05-26

**Authors:** Paola De Stefano, Elena Bianchi, Gabriele Dubini

**Affiliations:** Laboratory of Biological Structure Mechanics, Department of Chemistry, Materials and Chemical Engineering “G. Natta,” Politecnico di Milano, Italy

## Abstract

Drug discovery is an expensive and lengthy process. Among the different phases, drug discovery and preclinical trials play an important role as only 5–10 of all drugs that begin preclinical tests proceed to clinical trials. Indeed, current high-throughput screening technologies are very expensive, as they are unable to dispense small liquid volumes in an accurate and quick way. Moreover, despite being simple and fast, drug screening assays are usually performed under static conditions, thus failing to recapitulate tissue-specific architecture and biomechanical cues present *in vivo* even in the case of 3D models. On the contrary, microfluidics might offer a more rapid and cost-effective alternative. Although considered incompatible with high-throughput systems for years, technological advancements have demonstrated how this gap is rapidly reducing. In this Review, we want to further outline the role of microfluidics in high-throughput drug screening applications by looking at the multiple strategies for cell seeding, compartmentalization, continuous flow, stimuli administration (e.g., drug gradients or shear stresses), and single-cell analyses.

## I. INTRODUCTION

**FIG. 1.**
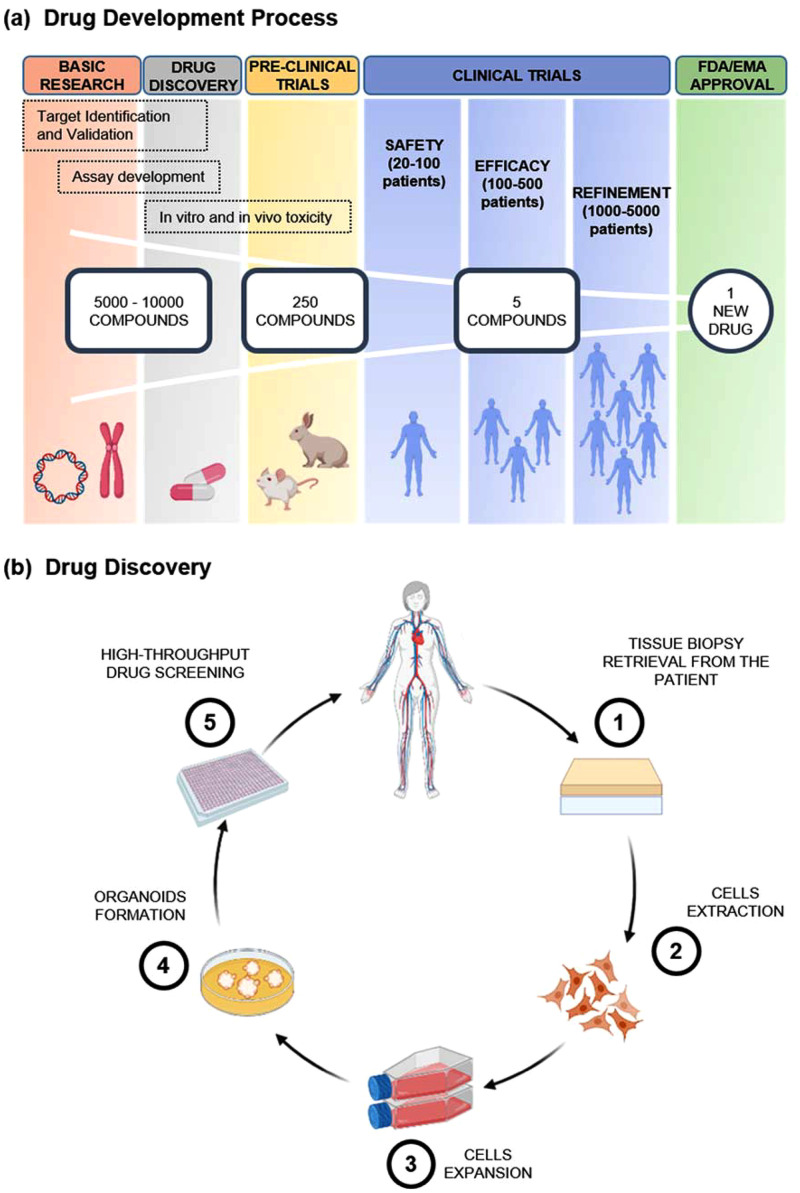
Top panel (a) shows drug development process divided in five main phases, including basic research, drug discovery, preclinical trials, clinical trials, and FDA/EMA approval. The first three phases are used for drug testing in non-human subjects to gather efficacy, toxicity, and pharmacokinetic information, while clinical trials are executed on human subjects. Phase I is done on healthy volunteers to test drug safety, side effects, best dose, and drug formulation method. Phase II is performed on larger groups once a dose or a range of doses are determined and is required to assess drug efficacy and side effects in a larger group of volunteers and patients. Phase III is to assess efficacy, effectiveness, and safety. Bottom panel (b) explains in detail the drug discovery stage, which encompasses five main steps: patient-derived cells are retrieved and extracted from tissue biopsies and then expanded in flasks following standard cell cultures protocols. Once obtained the required cell density, cells are detached from the substrate and resuspended in specific conditions, such as in Matrigel™, spinning bioreactors, or air–liquid interface method in order to form organoids. Finally, they are seeded in standard high-throughput multiwells and cultured with different drugs or combinations up to two weeks. Created with BioRender.com.

Drug development is an expensive and lengthy process (at least 12 years for a new drug to reach the market)^1^ (Fig. 1). On average, the cost of developing a new drug ranges between $1.3 and $2.8 billion, depending on the different disease areas and considering the developing phase (from discovery to market approval) and post-approval costs.^2,3^ This process can be divided into four main phases:

• Discovery and preclinical studies,
• Clinical studies,
• Regulatory review, and
• Post-marketing surveillance;

Among them, drug discovery plays an important role as only 2.5% of potential drug candidates reach preclinical studies and only 5–10 of them proceed to human testing.^4–6^ At this stage, large libraries of compounds (hundreds to thousands samples) are analyzed in high-throughput screening (HTS) assays with high purity and fully automatic robotic systems based on 384- and 1536-well plates in order to test metabolic function and pharmacokinetic and drug toxicity.^7–9^ During this process, several potential drugs are identified and optimized to determine therapeutic efficacy and potential risks.^10–12^

However, current HTS technologies—including modern robots, data processing and control software, liquid handling devices, and sensitive detectors to perform millions of biochemical, genetic, or pharmacological tests on samples in parallel—are expensive and are unable to dispense liquid volumes smaller than 1 *μ*l in an accurate and quick way. In addition, ongoing HTS approaches use costly biological samples and drug libraries and generally fail to reduce reagents’ consumption. In addition, further minimization of the well volume, as occurs when moving from 384- to 1536-well plates, is restricted by the quick and uncontrolled evaporation of the dispensed liquids,^9,13–15^ which compromises proper control of the concentrations.

Moreover, despite being simple, fast, and cost-effective with respect to animal studies, conventional 2D multiwell plate-based assays have reported significant perturbation in gene expression,^12^ as cells show different cell morphology, metabolic characteristics,^16^ and drug response.^17^ Indeed, they fail to recapitulate tissue-specific architecture and biomechanical cues.^16,18,19^ Therefore, the poor correlation between preclinical *in vitro* and *in vivo* data with clinical trials remains a major concern.^20^ The improvement and expansion of new systems for preclinical *in vitro* screening is becoming very important.^21^

One main cause for this failure is related to the static nature of current strategies, as they do not present any kind of perfusion and lack of shear stresses. Therefore, the culture conditions are too simple to mimic an *in vivo* microenvironment. To tackle this problem, bioreactors and even more the emerging field of organ-on-a-chip bear the potential to achieve a more organ- or tissue-specific dynamic culture by providing physical cues and improved nutrient supply.^22,23^ Indeed, organ-on-a-chip systems are micro-engineered devices that aim to resemble one functional unit of one living human organ. Specifically, they simulate an organ’s basic functions by exploiting similar physiological conditions. This allows the analysis of complex biological processes in response to several pharmacological stimulations. Furthermore, organ-on-a-chip systems can either host different cell lines, creating co-cultures, or be connected together to obtain the so-called body-on-a-chip systems, which are multiorgan platforms able to match functions of various organs.^24–26^

Moreover, although 2D cell cultures are still widely used and play a big role in this field, 3D cell cultures are progressively receiving more attention, as several types of cells need to grow in 3D systems to resemble more closely *in vivo* conditions, such as cellular events and disease progression.^27,28^

There are two major 3D culture systems: 3D scaffold-based^29,30^ and scaffold-free.^31^ In scaffold-based strategies, cells are seeded on prefabricated scaffolds or embedded in Extracellular Matrix (ECM)-like matrices before polymerization or solidification.^32^ In this case, scaffolds act as a support for cell adherence, growth, differentiation, and migration. Instead, in scaffold-free systems, cells are allowed to self-assemble in 3D constructs, like spheroids or organoids, without using any exogenous structures through cadherin-mediated adhesion.^33,34^ Among the 3D culture models, organoids are very attractive, as they represent cells aggregated in an organ-type, 3D specific matrix by avoiding cell attachment to the surrounding plate.

In particular, patient-derived organoids (PDOs) recapitulate histopathologic and genomic profiles of the tissue of origin, while maintaining genomic stability throughout passaging.^35–37^ However, given the complexity and specificity of 3D cellular constructs, fabrication still represents a challenge, as it requires specific setups. Moreover, despite their benefits, 3D models still suffer from several limitations.^38,39^ One of the major ones arises with the incorporation of multiple cell types resulting in more heterogeneity and data variability compared to 2D models. Moreover, 3D models lack standardization in size, geometry, volume, and density^40,41^ and, in the case of some naturally derived ECM matrices, exhibit batch-to-batch variations in biological properties.^38^ This limits the creation of large numbers of homogeneous organoids in formats compatible with high-throughput screening (e.g., 96- and 384-well plates), limiting the use of organoids in high-throughput drug screening applications.

Among the many methods developed to fabricate *in vitro* 3D tissue models, including hydrogel culture,^42,43^ hanging drop method,^44,45^ acoustic-based method,^46^ magnetic force,^47^ and bioprinting,^48,49^ microfluidics-based methods are among the most promising ones. Indeed, contrary to, for example, bioprinting, which fails to 3D print hydrogel bioinks into small-sized wells maintaining a spherical form due to well–bioink interactions, microfluidic devices can uniformly control large numbers of sized tumor spheroids or organoids by designing proper cell culture chamber geometries.^50,51^ Moreover, fabrication methods of microfluidic cell arrays can be easily tuned according to laboratories demands and assays purposes for both short-^12,52^ and long-term studies.^53^

Consequently, microfluidic devices offer the possibility of a technological breakthrough in modern HTS because of the above-mentioned peculiar characteristics.

In this Review, we want to further outline the impact of microfluidics in the implementation of high-throughput drug screening applications, by looking at the multiple strategies for cell seeding, compartmentalization, continuous flow, stimuli administration (e.g., drug gradients or shear stresses), and single-cell analyses.

## II. MICROFLUIDIC CELL SEEDING STRATEGIES

2D cell cultures have been widely used for decades due to several reasons, such as easiness to control a single well-defined cell type, simplicity in the manipulation of large quantities of cells, and the possibility to directly detect the cellular behavior.^54^ Furthermore, some cell types retain important phenotypic characteristics in monolayer cultures, such as epithelial cells, endothelial cells (ECs), muscle cells, and neural cells.^55^

**FIG. 2.**
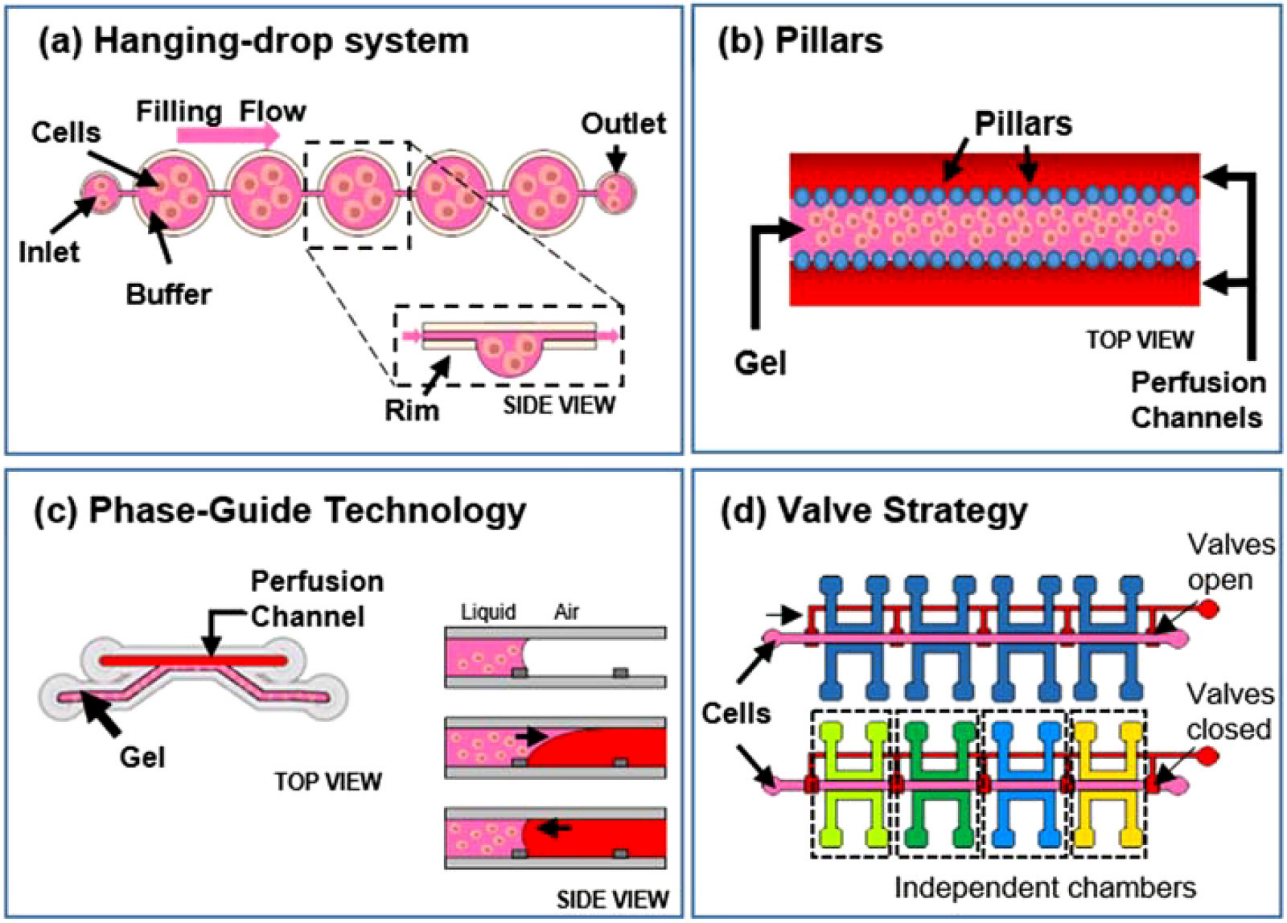
Microfluidic strategies for cell seeding, including (a) hanging drop approach, (b) and (c) pillars and phase-guide strategies, and (d) valving method.

Microfluidic cell culture systems can be adapted to both 2D and 3D culture methods. Many examples of 2D culture platforms use, for example, cell patterning techniques to modify surface properties for separating cells or positioning them in confined regions.^56,57^ Indeed, the laminar flow allows the deposition of multiple cells in microfluidic channels. However, other cell types need to grow in 3D systems to resemble more closely *in vivo* conditions. Therefore, during the last few decades, there was a fast growth of 3D cell cultures. Engineering culturing systems have rapidly evolved throughout the years trying to meet and surpass the standards of efficiency and throughput related to current systems. To this aim, microfluidics offers great advantages for manipulating cells and enabling, for example, spheroids formation. A common method widely used for obtaining scaffold-free cell cultures is the **hanging drop** approach [Fig. 2(a)]. In this case, after seeding, cells remain suspended in a drop of fluid hanging from a coverslip.

Tung *et al.*, for example, described a polystyrene 384-hanging drop array culture plate (16 rows and 24 columns), adaptable to already available HTS instruments. On the top, there is a plate lid in which every well corresponds to an access hole through which cells are seeded and medium is exchanged. At the bottom, instead, a 96-well plate is present filled with water coming from the reservoirs at the edges of the plate in order to avoid liquid evaporation.^58^ Marimuthu *et al.*, instead, presented a microfluidic platform for automatic multisize spheroid formation with constant volume hanging droplets starting from a single inlet loading at a constant cell concentration. This chip is composed of a series of pinholes structures connected through a channel network. On top of it, in correspondence to each pinhole, there is an array of cone-shaped wells (“funnel structure”) with increasing apex angles. During device filling, cells uniformly fill the funnels while pinholes act as a natural escape route for air bubbles. Once cells are seeded, they sediment into the hanging drop layer present below the funnel layer. This integrated platform is designed for multiple purposes, such as to form, treat, stain, and image multisize spheroids on-chip. Even if they use a 24-well plate for the proof-of-concept, the design can be easily generalized to a 96-well plate format.^59^

Alternatively, other strategies use microfluidics for transporting stimuli-responsive cell-laden hydrogels into pre-defined device regions confined by specific structures, such as **pillars** [Fig. 2(b)]^60–62^ **or phase-guides** [Fig. 2(c)].^61–64^ An example of this last technology is the commercially available OrganoPlate™ culture platform (Mimetas BV, the Netherlands). It is present on the market as two-lane or three-lane chip: a gel channel containing cells and one or two medium lines, respectively, for the two configurations.^65–68^ Thus, this technology enables free cell–cell interaction.

These are often implied in organ-on-a-chip devices, which are commonly characterized by a limited number of replicates; so they require massive parallelization and automation for use in high-throughput processes.

To address this limitation, several studies exploit the use of valves to isolate or fluidically connect multiple culture chambers according to the need [Fig. 2(d)]. For example, Visone *et al.* presented a chip composed of a layer containing multiple cell culture chambers and a valve layer separated by a thick polydimethylsiloxane (PDMS) membrane. According to the vacuum-pressure applied, the membrane could deflect downward opening valves and connecting culture chambers (active position) or it could close the channels maintaining them fluidically independent (rest position). In this way, only a single pipetting operation is required to obtain multiple independent 3D culture replicates within the same platform. Furthermore, even if they tested designs up to 96-chambers, the concept can be used to scale up the chip size and increase replicate numbers.^69^ Phan *et al.* described as well an arrayed version of an organ-on-a-chip platform that incorporates multiple vascularized micro-organs (VMO) on a 96-well plate. Each VMO is independently addressable and flow is driven through the micro-organ by hydrostatic pressure.^70^

However, cell samples are usually heterogeneous, so the overall drug response strongly depends on single-cell responses.^71,72^ Nonetheless, single-cell seeding is labor-intensive and often require robots. To overcome this limitation and reach high-throughput, single-cell trapping is a good alternative.^73,74^

## III. PERFUSION FOR CELL CULTURES

Another important aspect in *in vitro* models is perfusion. Indeed, dynamic conditions are strictly necessary to mimic physiological conditions as much as possible. Continuous flow is fundamental for cells as it allows nutrient supply and metabolic waste removal. Moreover, it can also be used to provide variable fluid shear stresses (FSSs), which are essential for proper cell evolution. Furthermore, as fluid volumes commonly range from nanoliters to milliliters, microfluidic systems represent a good solution to accomplish the task and also to provide drug concentration gradients. The implementation of dynamic conditions is basically consolidated in chips for a single sample, but it is challenging when applied with an HTS approach.

**FIG. 3.**
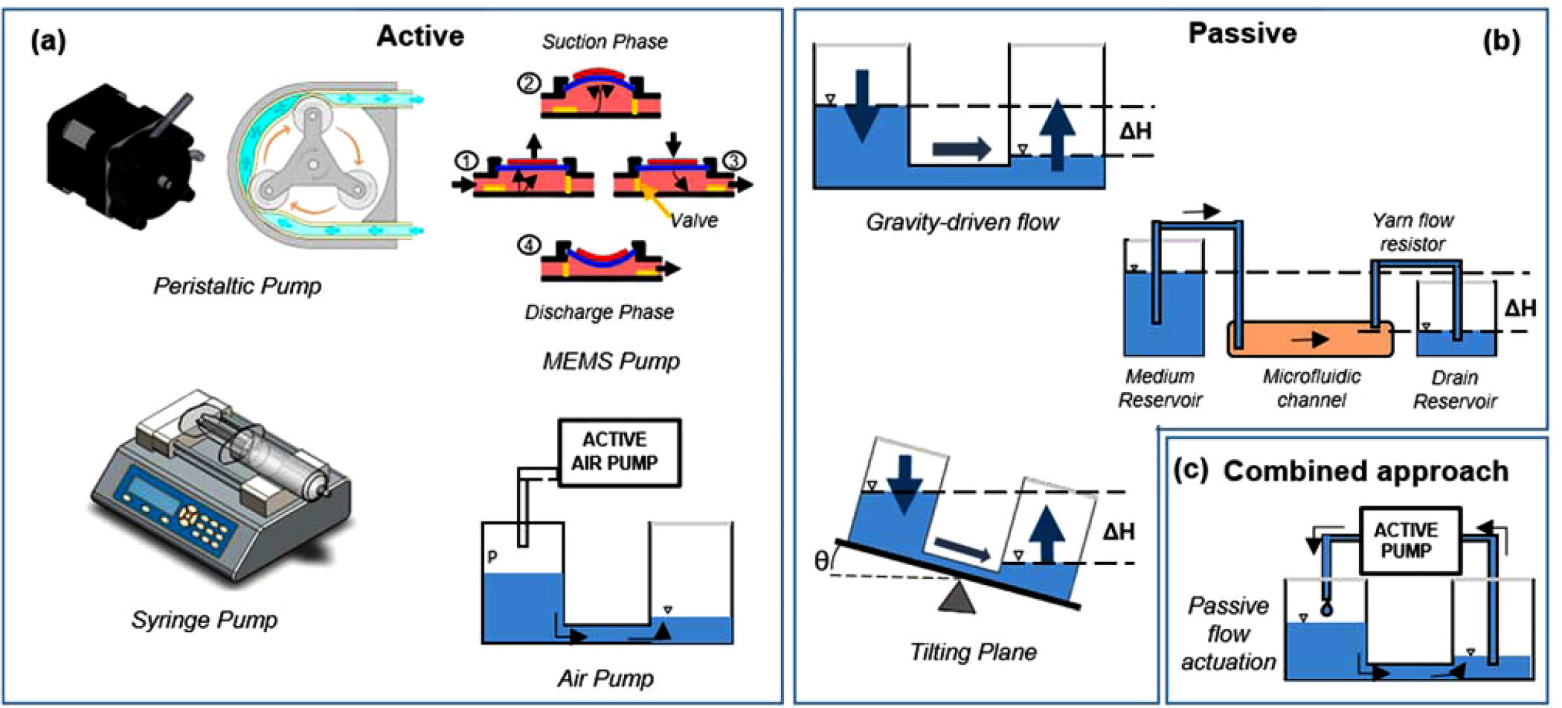
Strategies for pumping fluids into microfluidic chips. (a) Active methods include the use of big (syringe or peristaltic pumps) or miniaturized pumps (piezoelectric or pneumatically driven). (b) Passive strategies comprise tilting plane and hydrostatic pressure-driven systems. (c) Combined technology usually match passive and active approaches.

Fluid delivery systems can be either active (using external or integrated pumps) [Fig. 3(a)] or passive (exploiting gravity, surface tension or tilting plane solutions) [Fig. 3(b)].

### A. Active pumping systems

Many microfluidic devices use traditional external pumps to guarantee perfusion flow [Fig. 3(a)].^75,76^ **Syringe pumps** have been the most used devices in microfluidics, specifically for non-recirculatory flow applications, as they can deliver a small amount of fluids in continuous mode at a defined and precise flow rate.^61,62,77^

Wang *et al.*, for example, used this type of pump to induce neuronal differentiation by continuously administering 25 *μ*l/min of a specific culture medium. In this way, they were able to obtain long-term 3D organoid culture with a reduced fluid consumption.^61,62^ Similarly, Theobald *et al.* used a nEMESYS syringe pump to build a propulsion system. In this way, they were able to show that hepatic cells, grown in a simplified 3D liver-kidney-on-chip model under microfluidic conditions, abundantly and stably expressed metabolic-related biomarkers.^77^

In case of recirculatory and/or pulsatile flow, instead, the most common solutions are the **peristaltic roller pumps**. They exploit the peristalsis principle to pump a large variety of fluids. An internal rotor, which possesses a numbers of rollers at the extremities, compresses a flexible tube by constantly rotating. In this way, a body of fluid, trapped between rollers, is transported toward the pump outlet. The capability also for these pumps to manipulate small volumes of fluid makes them suitable for many microfluidic applications, such as organ-on-a-chip^78,79^ or body-on-a-chip.^24–26^ Mazzocchi *et al.*, for example, used a micro-peristaltic pump to let the medium continuously flow from an independent reservoir to discrete 3D patient-derived mesothelioma constructs.^79^

However, despite their benefits, both types of pumps are usually bulky. Therefore, it is very difficult to obtain handy and portable systems and to massively scale them up. Indeed, massive parallelization and automation are among the biggest challenges for HTS applications.

For these reasons, microfluidic applications often exploit micro-electromechanical (**MEMS**) **micropumps**, such as piezoelectric and pneumatically actuated pumps, as they are miniaturized, slim, and lightweight, and they can be easily integrated into microfluidic chips. Gómez-Sjöberg, for example, designed a fully automated cell culture screening system containing multiple pneumatic valves to deliver arbitrary culture media formulation to 96 independent 2D cell culture chambers.^75^

### B. Passive flow activation

Alternative to micropumps, passive systems have been adopted in HTS devices.

Passively driven microfluidic systems are rapidly increasing as the lab-on-chip field continues to grow. Their simplicity, compactness, and ease of use are key aspects when moving from small chips to HTS devices.

Passive pumping methods typically include pressure- and gravity-driven, capillary, hydrostatic, osmosis, vacuum-based, and surface tension methods [Fig. 3(b)].^76,80^ Among all these strategies, gravity-based passive systems are the most common as they use different heights of liquid reservoirs to achieve fluid flow from the higher to the lower one. This is very useful when a unidirectional flow is required, but often needs continuous refill of reservoirs.

Tilting platforms, instead, are able to overcome the refilling issue by exploiting the movement of a tilting plane to create a guided flow without using tubing or pumps. It has found application in the case of either unidirectional flow, thanks to the introduction of a bypass channel, or bidirectional flow.

For example, Lee *et al.* designed a microfluidic chip made of two different hydraulic resistance channels: a main perfusion channel and a bypass channel for medium recirculation. When the device is tilted in one direction, fluid moves into the perfusion channel and reaches the outlet. Otherwise, when the plane is tilted in the opposite direction, fluid rapidly flows backward through the bypass channel thanks to the smaller hydraulic resistance.^81^ In this way, the culture chamber is perfused by a unidirectional flow for most of the time (>99%). Even if they used this strategy for 2D cell culture, a similar mechanism can also be used for 3D models. Indeed, Esch *et al.* exploited the same mechanism for their 3D cell culture, by adding a passive valve to prevent backward flow using the capillary forces present at the air–liquid interface.^82^ Another example of tilting plate strategy with 3D models is reported by Eilenberger *et al.*, who designed a microfluidic multisize spheroid array to assess the effects of spheroid size on anticancer drug toxicity and compound penetration across an advanced model of the blood–brain barrier (BBB). They achieved continuous perfusion by gravity-induced bidirectional fluid circulation using an automated tilting motion of the microfluidic multisized spheroid array. Some advantages of this pumpless-flow strategy are the ability to adjust flow profiles by modifying the tilting angle and speed, reduce bubble formation, and reproduce pulsating nature of blood circulation.^83^

Another example of this strategy in HTS applications is reported by Jin *et al.*, who used a microplate array format of the microfluidic device (4 × 8) to model the hepatic tissue. In this case, fluid flow is simply generated using a rocker system. Moreover, they also added mixing channels to allow the generation of drug concentration gradients and also attempted to establish a multi-organ model by integrating other internal organoids.^84^

Another example of this method for bidirectional flow is presented by Petrosyan *et al.*, who exploited the commercially available three-lane OrganoPlate™ culture platform to recapitulate the 3D human glomerular filtration barrier in a high-throughput manner. By using a tilting plate, the platform achieves perfusion by moving fluids from one side reservoirs to the other side ones.^65^

As previously mentioned, the tilting plane strategy is only one of the possible passive pumping methods. Indeed, hydrostatic pressure or capillary-driven methods are also used in high-throughput applications.

Jung *et al.* presented a one-stop microfluidic device able to produce 3D lung cancer organoids in a size-controllable manner for testing drug sensitivity. They used a **yarn flow resistor** for siphoning off the medium in the range of a few hundred microliters per hour using a cotton yarn.^85^ Similarly, Jeong *et al.* exploited the same principle to measure 2D cell alignment and cytoskeletal arrangement of endothelial cells cultured in a microwell array inside the microfluidic channel.^86^

All these platforms are easy to use, low cost, and do not require complex tubing connections, so that dead volumes of metabolites and drugs are also significantly reduced.

As presented, both active and passive systems have several advantages and disadvantages. Therefore, to obtain further optimization of the existing pumping methods, it is also possible to combine them in a matched solution [Fig. 3(c)].

For example, the platform PREDICT-96 adapted and extended microphysiological systems (MPSs) to a 96-well plate configuration and integrated a novel, ultra-low volume, pneumatic-driven, recirculating pumping system, which enables media recirculation. Each unit has an active pneumatic pump that aspirates the medium from the outlet reservoir and pushes it toward the inlet. Then, medium flow in the MPS is achieved using media displacement between the two ports exploiting hydrostatic balance.^87^ By coupling active and passive mechanism, Tan *et al.* obtained a high-throughput microfluidic platform to recapitulate hepatocyte function. Similarly, Azizgolshani *et al.* exploited the same concept to build a high-throughput organ-on-chip platform for complex tissue modeling. They achieved two physiologically relevant flow regimes by designing an array of pneumatically driven, self-priming micropumps fluidically connected to the microfluidic culture plate (MCP), in which flow was determined by the hydrostatic balance between the chambers. As previously described for the PREDICT-96 platform, there is a coupling between active and passive pumping. Under steady-state conditions, the average flow rate generated in the MCP microchannels is equal to that of the micropumps. Pump flow rates are equal to the micropump stroke volume multiplied by the actuation frequency, which is set via a graphical user interface.^88^

Decoupling pumps from the chamber fluid flow has several advantages. First, it is possible to achieve complex systems while reducing encumbrance. Second, fluid flow is not directly dependent from the precise functioning of the pump. Finally, it is possible to achieve very small flow rates, even below the pump range.

## IV. STIMULI ADMINISTRATION METHODS FOR CELL CULTURES

### A. Shear stresses

**FIG. 4.**
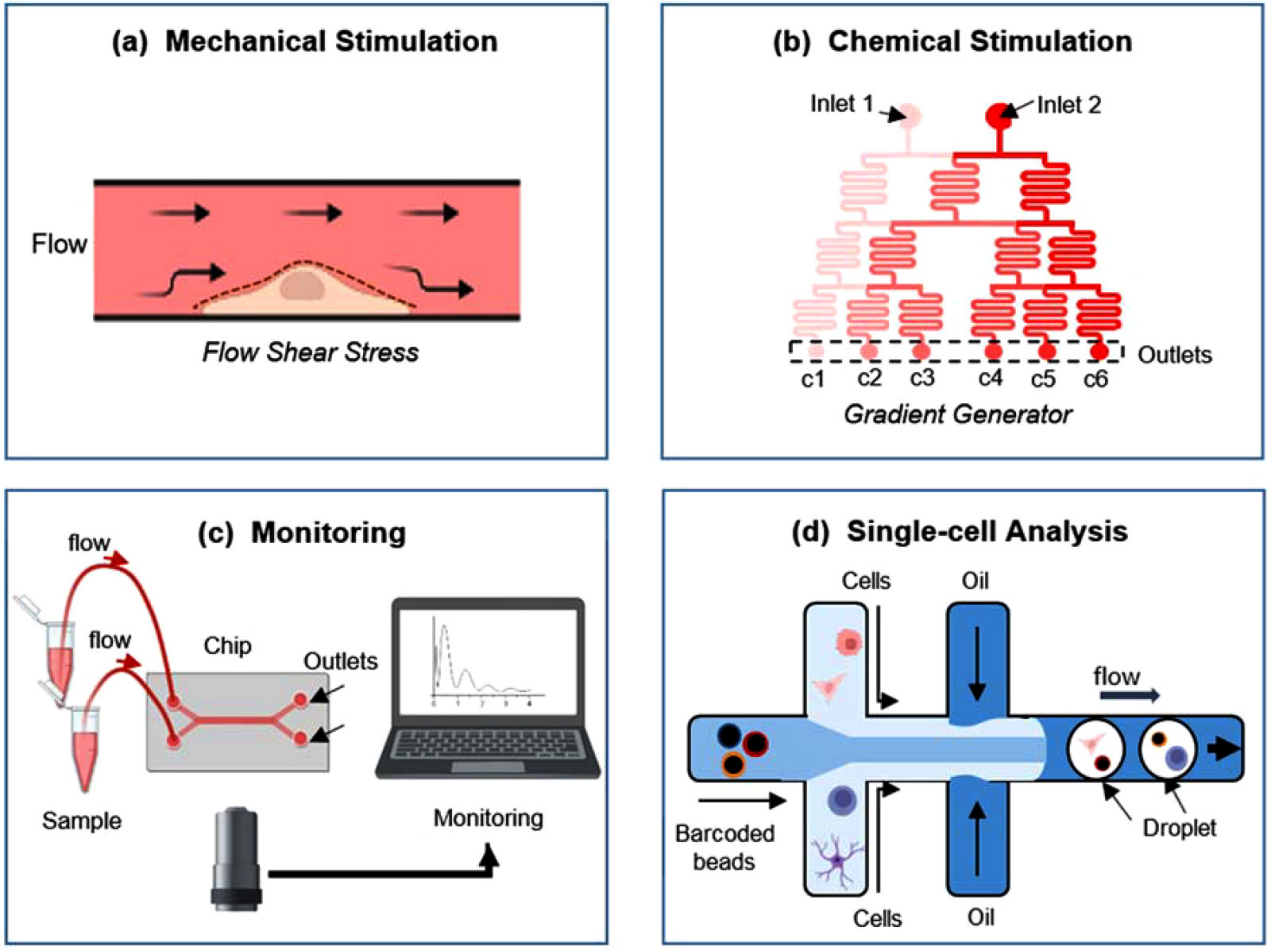
Examples of microfluidics in high-throughput applications, including (a) shear stress administration, (b) drug gradients production, (c) monitoring, and (d) single-cell analyses. Created with BioRender.com.

Physiological flow does not only provide energy transport and gas exchange but also provides mechanical stimulation, which is essential to regulate cells behavior (cell growth, morphology, proliferation, differentiation, adhesion, and migration)^89–92^ as cells adapt their response thanks to the various receptors present on the cell membrane [Fig. 4(a)]. Indeed, flows in the human body are characterized by different fluid shear stresses (FSSs) according to the organ and district under consideration.

Many diseases are exacerbated from abnormal FSS due to the rapid change of the microenvironment. Examples are thrombosis formation, related to elevated FSS in blood,^93^ or tumor metastasis induced by pathological fluid flows.^91^ Both endothelial and tumor cells are highly sensitive to FSS; even the low shear stress due to interstitial flow, although very small compared to the intravascular one, is able to stimulate some oncogenic signaling pathways or to promote vascular angiogenesis.^94–97^ Although the effects of hemodynamic fluidic shear stress on different cell types, such as endothelial cells and smooth muscle cells, have been extensively studied,^92,98,99^ the effect of fluidic forces on cancer cells is not yet clearly understood because of the diversity in tissue origin and drug-dependent sensitization mechanisms.^96^

In ovarian cancer, for example, Ip *et al.* found that cells, grown as 3D spheroids, significantly acquired the expression of epithelial-to-mesenchymal transition and cancer stem cell (CSC) markers and a remarkable chemoresistance to clinically relevant doses of frontline chemotherapeutic drugs cisplatin and paclitaxel when grown under fluid shear stress.^100^ Novak *et al.*, instead, by using a bioreactor capable of applying shear stress to cells within a 3D extracellular matrix, demonstrated that pulsatile shear stress promotes breast cancer cell proliferation, invasive potential, chemoresistance, and the urokinase-type plasminogen activator (PLAU) signaling. Finally, Au Ieong *et al.* revealed a distinct drug resistance between planar cell layers and 3D cell-spheroids by performing parallel screening on both of them.^101^

Overall, cancer cells experience two main types of fluid shear stress. The former one is related to the blood flow in the vascular microenvironment and the latter is generated by interstitial flow in the tumor microenvironment (TME). As previously stated, the velocity of the interstitial fluid is lower than that of intravascular fluid flow, yet it plays a crucial role in the growth and pathophysiology of cancer. For example, the rapid increase in the volume of solid tumors induces outward flow, and this flow changes the drug delivery path, thus reducing the efficiency of drug delivery.^102,103^ Furthermore, the flow reinforces the autocrine signaling in tumor cells, obtaining biochemical stimulation from the factors secreted by the cells themselves, and thereby stimulating directional invasion and migration.^153,154^ Interstitial shear stress can also upregulate TGF-β and activate fibroblast contraction to stiffen ECM.^97,104^

Therefore, proper evaluation of shear stress is strictly required when dealing with cancer cells, tumor metastasis,^105^ and drug resistance mechanism.^106^ Replicating the physiological features of the TME, such as fluidic shear stress, is essential to elucidate the dynamic response of tumor cells to biophysical and biochemical cues. In drug screening applications, it is crucial to properly mimic the *in vivo* microenvironment, as drug response can be altered by inappropriate stimulation.^107,108^ To this aim, platforms able to provide varying fluid shear stresses are strongly required to better mimic patho-physiological conditions. In this regard, microfluidic devices might offer a more rapid and cost-effective alternative to standard approach, as they present unique advantages for the development of efficient transport of drug carrier particles.^109^

Many studies in the literature focus on the impact of shear stresses. Lung epithelial cells stretched by breathing, intestinal cells exposed to peristaltic movement, or liver cells exposed to blood flow are only some other examples of key physiological forces occurring in the human body.^110^ The human intestinal Caco-2 cell line undergoes spontaneous villus differentiation when subjected to peristaltic movement instead of forming a 2D cell layer under static culture conditions.^111^ Blood flow on endothelial cells is another example of how specific microenvironmental features can modulate cellular function. Indeed, blood vessels are constantly exposed to hemodynamic forces in the form of cyclic stretch and shear stress due to the pulsatile blood flow. Endothelial cells (ECs) are able to convert mechanical stimuli into intracellular signals. Moreover, they use multiple sensing mechanisms to detect changes in mechanical forces between its luminal, abluminal, and junctional surfaces and its interior, including cytoplasm, nucleus, and focal adhesion sites.^92^

By looking at cytotoxicology tests, even inert drugs can be transformed by the liver becoming toxic at the local and/or systemic level.^112^ With this respect, blood flow exerting shear stresses on hepatocytes leads to changes in the expression of several genes.^113,114^ Moreover, when assessing drug nephrotoxicity, it should be taken into account that shear stress could induce several alterations to kidney epithelium: junctional reformation and cytoskeletal reorganization of renal proximal tubular epithelial cells,^115^ modifications in the expression of apical and basolateral transporters and sodium ions,^116,117^ or induction of active transport of glucose and amino acids across the epithelium.^118^ These changes in the physiological pathway induce modifications of all simple mechanisms of filtration, reabsorption, and secretion.

Considering the differences in the physiological flows throughout the human body, Feng *et al.* emphasized this aspect designing an integrated microfluidic chip. By using a syringe pump, they provided five steady FSS gradients ranging from 0.01 to 0.09 Pa, with specific volumetric flow rates. With this platform, they demonstrated that endothelial cells exhibited more intense effects of drug toxicity with respect to static conditions, outlining how fluid shear stresses enhanced drug toxicity. Furthermore, they also confirmed that vandetanib, an effective inhibitor targeting the vascular endothelial growth factor receptor (VEGFR) of endothelial cells, could exhibit clear dependency on toxicity.^107^

However, liver and kidney are not the only organs under investigation. Brain models are of particular interest for toxicological testing, as the brain structure and function are different from those of other organs in the human body. Indeed, the high structure complexity, caused by the presence of the blood–brain barrier (BBB), induces exclusion of up to 98% of small molecules.^119^ To this aim, *in vitro* models of the BBB are useful for the development of drugs capable of crossing this barrier. Moreover, differently from liver-on-a-chip models, which all have a fairly similar design, brain-on-a-chip models have different architectures depending on the mechanism under evaluation. Wang *et al.*, for example, developed a microfluidic BBB model to test whether shear stress is required to obtain *in vivo*-like barrier properties. The platform, composed of three layers, was designed without pumps or external tubing, thus considering only the blood residence time present in human brain tissues, mimicking *in vivo* BBB features for prolonged periods. By testing both large molecules and model drugs (caffeine, cimetidine, and doxorubicin), they demonstrated how the platform was able to replicate the physiologically measured permeability coefficients.^120^ Choublier *et al.* also investigated drug permeability of BBB under shear stress. They designed a device made of two communicating compartments via a semi-permeable membrane with 0.4 *μ*m pores, on which human cerebral microvascular endothelial cells (hCMEC/D3) were seeded. Each channel had an independent inlet and outlet, and the laminar flow was kept homogeneous for seven days. With their tests, they confirmed that hCMEC/D3 cells realign in the flow direction and demonstrated the use of their device for permeability studies.^121^ However, currently available chips may need further miniaturization and adaptation to be suitable for high-throughput screening in the context of preclinical testing.^110^

Looking at other systems, corneal *in vitro* models often lack the dynamic conditions related to eye blinking. To deal with this specific aspect, Abdalkader and Kamei developed a microfluidic platform enabling the dynamic culture of the human corneal barrier with recapitulation of eye blinking. The device consisted of upper and lower channels separated by a porous membrane on which human corneal epithelial cells (HCE-T) were seeded and cultured for ten days. Bidirectional and unidirectional flows were applied in the upper and lower channels, respectively, and the cells in the upper channels were stimulated with 0.6 dyn cm^−2^ shear stress. After 24 h, while the fluid stimuli did not affect cell adhesion, they facilitated the expression of cytokeratin 19 (CK-19) intermediate filaments in cells, indicating the strengthening of the barrier function. Furthermore, morphological single-cell analysis revealed an increase in the cell body area rather than nuclei.^122^

### B. Drug gradients

Compared with traditional drug screening approaches, emerging microfluidics-based drug screening systems are becoming powerful tools also to precisely control the extracellular chemical microenvironment, especially in high-throughput applications.^123–126^ Indeed, they are capable of testing different drugs or the same one at different concentrations and/or timings, still remaining time- and cost-saving. Moreover, its high resolution at the small scale allows generating spatiotemporal chemical gradients, which are crucial for mimicking *in vivo* microenvironment, and especially to analyze diseases like tumorigenesis^127,128^ [Fig. 4(b)].

To achieve this goal, there are several well-known strategies, including convection flow, diffusion, droplet, surface modification, flow switching/shifting, and integrated valves/pumps, already well detailed in the review by Chen *et al.*^127,129^ Overall, they solved many issues related to the complexity of the extracellular microenvironment, but they still need further upgrades in terms of integration, multiplexing, and precision in order to increase the throughput. Indeed, as above-mentioned, the extracellular microenvironment is not limited only to one specific stimulus, but it is a combination of chemical, mechanical, and electrical perturbations. In fact, it is well-established how mechanical or electrical stimuli can also affect cell response to drug stimuli by affecting, for example, cell membrane permeability. To tackle this aspect, Yahyazadeh Shourabi *et al.* developed an integrated concentration gradient generator capable of providing cell monolayers with both mechanical and chemical stimuli. Their chip consisted of two PDMS layers bonded together with a membrane interposed and integrated with two bubble traps. Thanks to this design, they were able to provide four different drug concentrations, adjustable shear stresses, and osmotic pressure gradients.^130^ On the contrary, Sun *et al.* wanted to analyze the effects of a number of drug concentrations while maintaining minimum shear stress. For this purpose, they integrated an on-chip cell culture with three laminar flow diffusion channels. In this device, there were 42 independent cell culture chambers, in which cells could be incubated with minimal shear stress thanks to a series of micropillars.^131^

Moreover, when dealing with high-throughput applications, it is essential to have microfluidic chips integrated with sensors and/or readout systems. Zhang *et al.*, for example, designed a “Christmas Tree mixer” integrated with a custom software for fast readout and data analysis. Thanks to this system, they generated a logarithmic concentration mixing ratio between drug pairs and were able to screen 172 different treatment conditions over 1032 3D cancer spheroids.^132^ Alternatively, Gao *et al.* and afterward Liu *et al.* integrated a microfluidic device, comprising a multiple gradient generator, a microscale cell culture chambers array, and an on-chip solid-phase extraction columns, with an online electrospray ionization quadrupole time-of-flight mass spectrometer. In this way, drug absorption and cytotoxicity evaluation could be simultaneously performed.^133,134^

## V. MONITORING

As evidenced by all the described devices, the overall complexity of the chips can be tailored according to the needs. Simple devices integrated only pumps or valves, while more sophisticated ones integrated sensors to simultaneously and continuously monitor culture environment, cell behavior, or cell stimulation.^135^ Therefore, integration of microfluidics with online or offline bioanalytical instruments is essential, especially in high-throughput applications [Fig. 4(c)].

Cell culture parameters are the major determinants in 3D organoids formation.^135^ For this reason, many studies focus on the integration of electrochemical sensors. Weltin *et al.* proposed a multiparametric microphysiometry system for the dynamic online monitoring of human brain cancer cell metabolism. The chip was fully integrated with chemosensors for pH and oxygen monitoring and biosensors to quantify lactate production and glucose consumption.^136^ Zhu *et al.* developed a versatile device for multianalyte quantification. The analytes were analyzed into innerbuilt channels containing screen-printed electrodes, modified with different nanomaterials and sensing agents. These electrodes, located at different positions in the chip, provided specific responses to the corresponding indicators.^137^

Monitoring cell behavior is as equally important as cell culture media evaluation. Jeong *et al.* integrated the transendothelial electrical resistance (TEER) in a 4 × 4 intersecting microchannel array to allow label-free real-time analysis of the blood–brain barrier, crucial for the development of therapeutics against neurological diseases.^138^ Alternatively, Asif *et al.* presented a theranostic proximal tubule-on-a-chip model for live monitoring. TEER electrodes, an optical pH sensor and a microscope were integrated into the platform for the real-time monitoring of the cell adhesion and culture medium pH.^139^

However, human organ functions strongly depend on mechanical, chemical, and electrical stimulations. Therefore, it is extremely important also to monitor the stimuli provided to cells. Mechanical deformation is crucial, for example, for mimicking lung^140^ or electrical stimulation for brain^141^ or heart.^142^ Qian *et al.* presented a novel cardiac platform that can record cardiac tissue adhesion, electrophysiology, and contractility on the same chip. The device has a microelectrode array for field potential readouts and an interdigitated electrode array for impedance readouts. Thanks to these two different sensor arrays, the authors achieved real-time, non-invasive data acquisition of both cardiac electrophysiology and contractility.^143^

All those aspects are even more important when looking at multiorgan-on-chips (MOC) platforms. Zhang developed a fully integrated MOC together with modular physical, biochemical, and optical sensing units, operated in an automated manner.^144^

## VI. SINGLE-CELL ANALYSES

The creation and maintenance of a cellular microenvironment as well as monitoring of cell response is very important. However, cell heterogeneity is particularly difficult to be investigated with standard approaches^145^ as it hinders the accurate modeling of diseases due to many reasons, like the interpenetration of biomarkers levels and patient responses to specific therapies.^146^

Moreover, conventional techniques, such as flow cytometry or single-molecule fluorescence spectroscopy, are low-throughput.^147^ To this aim, droplet microfluidics has revolutionized single-cell analysis field thanks to the capability to compartmentalize cells within picoliter droplets, also allowing the discovery of rare cells in tumors or in the immune system.^148^ It is a two-phase microfluidics in which two immiscible liquids are forced to merge forming plugs of one fluid within a carrier fluid [Fig. 4(d)]. This strategy allows us to significantly reduce the reaction volume and to physically and chemically isolate droplets from each other. Droplet formation can be achieved in a highly repeatable manner using device geometries such as T-junctions, flow focusing, and co-flow.^148^

The possibility to study tumor heterogeneity at the single-cell level is even more attractive in cancer research where understanding molecular, cellular, genetic. and functional heterogeneity is still one of the major challenges.^149^ For this reason, single-cell approaches are widely used for cancer biology, diagnosis, and therapy. When dealing with drug screening applications, the evaluation of drug cytotoxicity as well as multidrug resistance is even more important. Moreover, it is particularly advantageous in precision medicine considering the limited availability of tumor samples. Indeed, Wong *et al.* exploited this strategy and developed a 2.4 × 2.4 cm PDMS chip to perform drug screening on both cancer cell lines (suspended and adherent) and cells dissociated from human primary tumors. Single cells were dispersed in aqueous droplets and imaged within 24 h of drug treatment to assess cell viability by ethidium homodimer 1 staining. Results showed that five conditions could be screened for every 80 000 cells in one channel on the chip. The versatility in the screening conditions was seen as an example for analyses with other types of cancers. Even if it was still a low-throughput tool, it was a starting point for further technological advancement to cut down sample size in advent to personalized cancer therapy.^150^ Alternatively, Du *et al.* developed an oil-covered two-dimensional droplet array chip based on sequential operations. In this system, the chip was coupled with a syringe pump connected with a tapered capillary, and it was automatized to achieve complex parallel multistep operations, such as long-term cell culture, medium changing, schedule-dependent drug dosage and stimulation, and cell viability testing. In this way, the authors were able to reduce 10- to 1000-times drug consumption for each screening test compared with traditional drug screening.^151^ Looking at high-throughput systems, Kulesa *et al.* proposed another device to predict synergy between more than 4 000 investigational and approved drugs and a panel of ten antibiotics against *Escherichia coli*. Their device formulated a chemical library in nanoliter droplet emulsions and automated the construction of chemical combinations *en masse* using parallel droplet processing. They were able to even find a range of drugs not previously indicated for infectious disease that synergize with antibiotics, such as vancomycin, erythromycin, and novobiocin.^152^

Moreover, it is important to study also monoclonal antibodies, which play a crucial role in drug discovery. Nowadays, it is still challenging to screen them due to the binding of cell-surface receptors or the binding to target cells rather than purified proteins. For this reason, Shembekar *et al.* presented a high-throughput droplet microfluidic approach employing dual-color normalized fluorescence readout to detect antibody binding. This enabled them to obtain quantitative data on target cell recognition, using very reduced quantities of IgG per assay. In this way, individual clones secreting specific binders were enriched 220-fold after sorting 80 000 clones in a single experiment.^153^

## VII. TECHNOLOGICAL LIMITATIONS AND FUTURE PERSPECTIVES

As presented, microfluidics can be considered a potential tool at each phase of the drug discovery process, such as cell seeding, perfusion, and drug administration as well as single-cell analysis. Microfluidics can allow high-throughput and multiplexed drug screenings at cell, organ, and whole-body levels. However, microfluidics and HTS applications have always been considered at opposite poles with an unbridgeable gap between them due to a number of microfabrication constraints. Indeed, the majority of microfluidic chips is realized using PDMS, as it is biocompatible, transparent, permeable to gas, able to replicate structures up to the nanolevel scale and has a low autofluorescence. Moreover, the possibility to use it with soft lithography methods allows the rapid production of prototypes.^154^ However, it is well-known that PDMS has adverse effects on cell-based experiments, such as molecules absorption, inherent hydrophobicity, swelling behavior in common solvents, water evaporation, sensitivity to some chemicals, and the possibility to release incomplete reticulated PDMS.^155^ Moreover, mass production of microfluidic devices cannot be achieved by current PDMS-based techniques due to the difficulty in scaling up the manufacturing process.

Furthermore, the standard material for cell culture in biology is polystyrene (PS), which is the most common material for laboratory cultureware. Additionally, all validated research conclusions in cell behavior and function were achieved with standard PS plates, which introduces a further obstacle when moving toward biology research.^156^ Therefore, PS has the potential to help bridge the current gap between the state-of-the-art in microfluidics technology and the needs of biologists. However, the translation is hardly feasible due to several reasons, such as higher costs of thermoplastic fabrication compared to soft lithography, in case of low volume production due to the use of molds resistant to high temperatures and pressures. Indeed, there are two traditionally methods used to produce plastic devices: injection molding and hot embossing. Kim *et al*., for example, designed and fabricated a high-throughput injection molded microfluidic device that enabled real-time chemical profile control for single-cell analysis. They optimized channels dimensions according to the microfabrication process while retaining the desired function. By looking at their results, they were able to generate diverse stimulation profiles and to obtain identical functionality compared to previous researches.^157^ While injection molding is reliable, reproducible, and low cost in high volume production cases, hot embossing is more accessible and flexible for low to medium production and is less expensive to set up, making it the most practical choice for academic labs. However, in both cases, molds are fabricated by either computer numerical control (CNC) or precision laser machining, which can be time-consuming, laborious, and can affect surface roughness, interfering with optical microscopy and cell imaging. To solve this issue, molds need to be polished, but this increases costs. Alternatively, recent works have begun to demonstrate more practical, low-cost methods for making molds, such as using high strength epoxies instead of metal molds.^158,159^ PS can also be directly embossed from a positive-relief PDMS mold, which takes advantage of the compliance of PDMS to facilitate demolding.^160^ These methods only add one extra step to the mold fabrication process and do not require the use of additional equipment beyond a heated press or hot plate. However, it is still difficult to produce very complicated device structures or to exploit pneumatic valve mechanism to obtain complicated functions.

## VIII. CONCLUSION

In conclusion, microfluidics may offer a more rapid and cost-effective alternative to standard approaches. Up to now, simple models with reduced number of culture chambers are almost consolidated, while high-throughput devices still need further implementation. Since the existing systems and preclinical models are inefficient for predicting clinical outcomes, it could be extremely important to exploit this technology to build more sophisticated systems, even if the number of cell culture chambers would be less than standard high-throughput culture plates. Indeed, microfluidic devices will be able to more closely resemble *in vivo* conditions while reducing significantly the amount of reagents needed for drug screening tests.

## Data Availability

Data sharing is not applicable to this article as no new data were created or analyzed in this study.
